# Non-Heme Iron Absorption and Utilization from Typical Whole Chinese Diets in Young Chinese Urban Men Measured by a Double-Labeled Stable Isotope Technique

**DOI:** 10.1371/journal.pone.0153885

**Published:** 2016-04-21

**Authors:** Lichen Yang, Yuhui Zhang, Jun Wang, Zhengwu Huang, Lingyan Gou, Zhilin Wang, Tongxiang Ren, Jianhua Piao, Xiaoguang Yang

**Affiliations:** 1 Key laboratory of Trace Element Nutrition of the Ministry of Health, National Institute of Nutrition for Health, Chinese Center for Disease Control and Prevention, Beijing, 100050, P.R. China; 2 Bethune Military Medical College, Shijiazhuang, Hebei, 050081, P.R. China; 3 National Institute of Metrology, National Research Center for Certified Reference Material, Beijing, 100029, P.R. China; National Institute of Agronomic Research, FRANCE

## Abstract

**Background:**

This study was to observe the non-heme iron absorption and biological utilization from typical whole Chinese diets in young Chinese healthy urban men, and to observe if the iron absorption and utilization could be affected by the staple food patterns of Southern and Northern China.

**Materials and Methods:**

Twenty-two young urban men aged 18–24 years were recruited and randomly assigned to two groups in which the staple food was rice and steamed buns, respectively. Each subject received 3 meals containing approximately 3.25 mg stable ^57^FeSO_4_ (the ratio of ^57^Fe content in breakfast, lunch and dinner was 1:2:2) daily for 2 consecutive days. In addition, approximately 2.4 mg ^58^FeSO_4_ was administered intravenously to each subject at 30–60 min after dinner each day. Blood samples were collected from each subject to measure the enrichment of the ^57^Fe and ^58^Fe. Fourteen days after the experimental diet, non-heme iron absorption was assessed by measuring ^57^Fe incorporation into red blood cells, and absorbed iron utilization was determined according to the red blood cell incorporation of intravenously infused ^58^Fe SO_4_.

**Results:**

Non-heme iron intake values overall, and in the rice and steamed buns groups were 12.8 ±2.1, 11.3±1.3 and 14.3±1.5 mg, respectively; the mean ^57^Fe absorption rates were 11±7%, 13±7%, and 8±4%, respectively; and the mean infused ^58^Fe utilization rates were 85±8%, 84±6%, and 85±10%, respectively. There was no significantly difference in the iron intakes, and ^57^Fe absorption and infused ^58^Fe utilization rates between rice and steamed buns groups (all *P*>0.05).

**Conclusion:**

We present the non-heme iron absorption and utilization rates from typical whole Chinese diets among young Chinese healthy urban men, which was not affected by the representative staple food patterns of Southern and Northern China. This study will provide a basis for the setting of Chinese iron DRIs.

## Introduction

Iron deficiency, mainly caused by low iron intakes or absorption, is one of the most important nutritional problems worldwide, particularly in developing countries. Iron deficiency anemia affects approximately one-quarter of the world’s population [1]. Meanwhile, iron overload has also become a prominent public health issue. A national report showed constant high prevalence (>30%) of iron overload (ferritin concentration >200 μg/L) between 1999–2002 in U.S. men of 20 years and older [2]. A survey of the year 2005 in Beijing and Shanghai of China indicated that 10.6% of men (ferritin concentration > 300 μg/L) and 14.7% of women (ferritin > 200 μg/L) exhibited iron overload [3]. As the body lacks of active iron excretion mechanisms and thus human body iron balance is regulated by absorption [4], high iron intake and /or stores are reportedly associated with increased risks of chronic diseases such as pre-diabetes, chronic ambulatory peritoneal dialysis and colorectal cancer [5–7]. Therefore, it is important to correctly plan the iron intake in diets.

To help people better plan diets and/or evaluate nutrient status of individuals and groups, dietary reference values [4, 8] and dietary reference intakes (DRIs) [9–15] have been suggested in Europe, USA, Canada, East Asia countries, and in China. The present DRIs in China [15] were mainly based on a 2000 Chinese Total Diet Study (unpublished data). With the recent progress in the related fundamental studies and update of the data relating to the national dietary status, the values for DRIs need to be accordingly adjusted. Therefore, DRIs should be updated in China to more accurately reflect the requirement of nutrients of Chinese population. For the setting of iron DRI, it is essential to accurately estimate the bioavailability of dietary iron. However, currently there lacks of available data that can be used directly for the setting of Chinese iron DRIs. Dietary iron comprises heme iron (mainly from animal tissues) and non-heme iron (mainly from plant foods). Compared with the heme iron, non-heme iron generally has much lower bioavailability [16], suggesting that the determination of non-heme iron absorption and utilization is key for the setting of iron DRIs.

The absorption of iron may differ significantly with various diet- and host-related variables, including the chemical forms of the nutrients and means of cooking and processing (diet-related), presence of enhancers and inhibitors of iron absorption (diet-related), life-stage, and nutritional and health status (host-related) [17, 18]. It was reported that iron absorption from typical Latin American diets ranged from 7.5% to 13.4% [19], and that the overall iron bioavailability from a mixed American or Canadian diet was 18% [10]. WHO/FAO proposed iron bioavailability of 15%, 12%, 10%, or 5%, depending on the dietary composition and other factors [20]. Studies from the United States, Europe and Mexico revealed large variations in mean non-heme iron absorption (0.7–22.9%) from whole diets [21–25], indicating there lacks agreement in relation to non-heme iron absorption both between and within countries. In addition to dietary patterns, there are big genetic differences between the Chinese population and those from other countries. For example, the incidence of the HFE gene mutations contributing to hemochromatosis as a disorder that causes excess high iron absorption [26, 27] is low in Chinese Han population (major Chinese population) than in white populations [28–30]. Therefore, it is difficult to directly refer to related iron absorption data from foreign studies. In addition, since the iron absorption depends on iron status, dietary enhancers and inhibitors, etc., extrapolation of the present findings to other countries such as China will require additional high-quality controlled trials [18]. It was therefore recommended that domestic data should be preferred when setting the iron DRIs in different countries. Moreover, the iron absorption data from single meals is often different from whole diets, which cannot be correctly used to estimate dietary iron absorption. The iron absorption data should be obtained from the whole diets.

The stable isotope labeling technique is currently the most accurate method for determining dietary mineral absorption and utilization through the oral administration of a single isotope or two isotopes (i.e. one given orally and the other intravenously [31–33]. The primary aim of this study was to investigate the non-heme iron absorption and biological utilization from typical whole Chinese diets in young Chinese healthy urban men using a double-labeled stable isotope technique, and thus to generate some data base from which bioavailability values could be derived for setting iron DRIs for the Chinese population. In addition, considering China is a large country where the dietary patterns vary among the different parts, this study was also to observe if the non-heme iron absorption and utilization could be affected by the staple food patterns of Southern and Northern China. This study will provide a basis for the setting of Chinese iron DRIs.

## Materials and Methods

### Subjects

Twenty-two eligible young Chinese healthy urban men (11 from Southern China and 11 from Northern China) aged 18–24 years were randomly recruited between January 2010 and March 2011 from Bethune Military Medical College (Shijiazhuang, Heibei, China). Subjects did not take any vitamins or mineral supplements at least one month before the experiment. Subjects with diseases (such as malabsorption and ulcer) that could affect the iron absorption or with chronic or acute inflammatory diseases, and those regularly taking medication that could affect iron absorption were excluded. In particular, accurate examinations were conducted to exclude subjects with abnormal iron nutrition statuses and potential inflammation.

Rice and wheat belong to the most consumed cereals in the world, and rice and wheat-prepared buns are also major staple foods in China. On the basis of respecting their conventional dietary inhabits as possible and obtainment of their agreement, 22 eligible subjects were then randomly assigned to rice group (n = 11) and steamed buns group (n = 11), taking rice and steamed buns as the staple food, respectively. The trial was registered in the Chinese Clinical Trial Registry (No: ChiCTR-TRC-09000580). This study was approved by the Ethics Committee of the National Institute of Nutrition and Food Safety, Chinese Centers for Disease Control and Prevention (Beijing, China), and written informed consent was obtained from all subjects prior to participation.

### Study design

**Fig 1:**
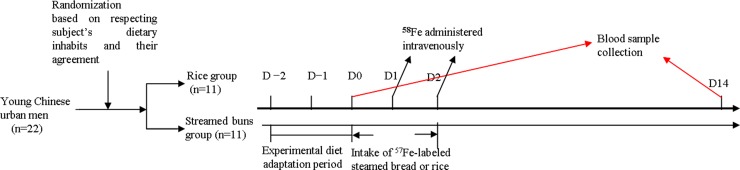
Experimental diagram.

The study flow chart is shown in Fig 1. First, the subjects began a 2-day experimental diet adaptation period. During this period, subjects were observed if they could accept the designed dietary and the related processing methods. In some cases, in response to the feedbacks from the subjects, fruit types and snacks would be added. Meanwhile, the subjects were further observed the dietary habits and healthy conditions, and were also asked to fulfill some related questionnaires such as previous diseases and family history. On day 0, a baseline venous blood sample was drawn after an overnight fast to determine the serum levels of haemoglobin (Hb) and C-reactive protein (CRP), and other iron status indexes (i.e. unsaturated iron-binding capacity [UIBC], serum iron [SI], soluble transferrin receptor [sTfR], and serum ferritin [SF]). The baseline isotopic composition was also analyzed. During the experimental diet, the subjects received 6 test meals (i.e., iron-fortified steamed buns or rice) labeled with ^57^FeSO_4_ over 2 days (approximately 3.25 mg/day; isotope ratio at breakfast, lunch and dinner was 1:2:2) under standardized conditions and close supervision.

One hour after dinner in each experimental day, 2 mL ^58^FeSO_4_ aqueous solution was placed into a 250-mL infusion bag with 0.9% saline, which was infused into subjects (approximately 2.4 mg/day) slowly over 50 min. Fourteen days later, a second venous blood sample was drawn from each subject after an overnight fast. Non-heme iron absorption and utilization were estimated.

### Experimental diet

The varieties and quantities of the foods in the experimental diet were determined on the basis of the typical representative diet from the latest China National Nutritional Survey [34] and cluster analysis. Foods were classified into eight categories: grains, beans and bean products, vegetables, fruits, meat, seafood, dairy products, and eggs. According to the food patterns reported by the subjects and market supplies, a 2-day menu (Table 1) was designed and repeatedly served to each subject throughout the experimental period. The food materials were purchased from the local market. The amounts of all foods served each day were accurately weighed and uniformly prepared according to the principles of nutrient and energy balance.

**Table 1:** Menu for the experimental period.

		Day 1	Day 2
**Breakfast**	**Staple food**	Steamed buns/rice	Steamed buns/rice
	**Non-staple food**	Milk	Milk
		Tomato and egg soup	Egg and cucumber soup
		Green pepper with shredded potato	Stir-fried cabbage
		Seaweed strip salad	Seaweed strip salad
		Tofu salad	Celery salad
**Lunch**	**Staple food**	Steamed buns/rice	Steamed buns/rice
	**Non-staple food**	Stir-fried chicken breast	Stir-fried squid
		Stir-fried shredded pork tenderloin	Stir-fried shredded pork tenderloin
		Vegetarian stir-fried green beans	Braised eggplant
		Vegetarian stir-fried cabbage	Stir-fried green pepper
		Seaweed and bok choy soup	Seaweed and bok choy soup
**Dinner**	**Staple food**	Steamed buns/rice	Steamed buns/rice
	**Non-staple food**	Braised ribbonfish	Stir-fried shrimp
		Braised beef	
		Stir-fried mushroom	Stir-fried tofu
		Stir-fried cabbage with vinegar sauce	Stir-fried cauliflower
		Seaweed and spinach soup	Seaweed and spinach soup
		Apple	Pear

### Preparation of isotopically labeled iron

Isotopic labels (^57^FeSO_4_ and ^58^FeSO_4_) used in this study were prepared in solution from ^57^Fe- or ^58^Fe- enriched elemental iron (Isoflex, San Francisco, CA, USA) by dissolution in 3M H_2_SO_4_ and dilution to appropriate concentration with ultrapure water. The stable iron isotope plus 10 mg ascorbic acid, a type of enhancers of iron absorption from diets [25, 35], per mg iron was placed in ampoules. The ^58^Fe preparation for intravenous infusion was sterilized and assessed for pyrogens. The exact isotopic composition of the stable isotope labels was measured using multiple collector inductively coupled plasma mass spectrometry (MC-ICP-MS, GV instrument, UK). The final concentrations of ^57^FeSO_4_ and ^58^FeSO_4_ were analyzed by a PinAAcle 900 T atomic absorption spectroscopy (PE, Shanghai, China). The administered dose of ^58^FeSO_4_ was determined by accurate weighting of the ampoules for each test meal before and after addition of the stable isotope solution.

### Test meal preparation

Steamed buns labeled with ^57^Fe were prepared for each subject separately. The same total dose of ^57^FeSO_4_ (approximately 3.25 mg/day) for each subject was thoroughly mixed with a specific weight of flour. The mixture was then combined with active dry yeast and an appropriate amount of triple-distilled water to form dough. The dough was kneaded to make steamed buns in a normal manner and placed into marked steamers. The buns were allowed to rise fully, steamed for 30 min, removed and allowed to cool to room temperature, packed in individual sealed vacuum packages, marked, and placed in a -20°C freezer for later use.

Prior to each meal, the ^57^Fe-labeled steamed buns were removed from its packaging and placed in the steamer. After 15 min of steaming, the labeled buns were distributed to the corresponding subjects (for each subject, one bun was for breakfast and two for lunch and dinner, respectively). All the ^57^Fe-labeled buns were required to be consumed completely.

Similarly, rice labeled with same dose of ^57^FeSO_4_ as bun group was also prepared for each subject in rice group per day. The total ^57^Fe preparation dose for each test subject (approximately 3.25 mg/day) was added to some appropriate amount of rice and water in some specific glass box. At the early morning, all the rice for that experiment day would be steamed and ready for all the rice-group participants. The amount ration consumed by breakfast: lunch: dinner was 1:2:2. After breakfast, the prepared ^57^Fe rice was reserved in refrigerator and heated before lunch and diner. Each subject was required to finish every rice grain at every meal as well as an entire bowl of soup.

For the subjects, other streamed buns or rice without labeling could also be chosen if they needed to eat more staple foods.

### Dietary sample collection and detection

Each subject’s intake of every type of food at each meal throughout the experiment was accurately measured and recorded. Duplicate portions of meals provided during the experimental period were simultaneously collected, homogenized with a mixer, packed, and stored at −20°C. Common dietary nutrients (including calories, zinc, carbohydrate, fat, protein, dietary fiber, etc.), and enhancers (ascorbic acid) and inhibitors (phytic acid, calcium) of iron absorption [4, 25, 35, 36] were measured using the related routine protocols of the National Standard of the People’s Republic of China. Particularly, for the measurement of phytic acid, briefly, after the collected food was homogenized, phytic acid in the homogenate was purified by anion exchange resin, eluted by sodium chloride, and reacted with ferric trichloride-sulfosalicylic acid mixture to show color-fading reaction. The phytic acid content could be determined by the measurement of the absorbance at 500 nm using a spectrophotometer through a standard work curve.

### Blood analysis

Serum was separated from whole blood by centrifugation and frozen at −20°C with no freeze–thaw cycles. Hb, SF, CRP, sTfR, SI, and UIBC levels were measured with an IMMULITE automatic system (Roche Diagnostics GmbH, Mannheim, Germany). Iron deficiency was defined as an SF level < 30 μg/L according to the reference range of the kit, and early iron deficiency was determined as sTfR >4.4 mg/L [2]. Iron deficiency anemia was defined as SF < 30 μg/L and Hb < 130 g/L. CRP was expected to remain at normal levels (<5 mg/L) according to the reference range of the Kit.

Blood samples were mineralized with an HNO_3_/H_2_O_2_ mixture and microwave digestion using a microwave digestion system (EXCEL, Shanghai, China), which was followed by separation of the sample iron from the blood matrix with ion exchange method. Fe isotopic analysis was performed by MC-ICP-MS with standard-sample bracketing method. The standard used to correct the instrumental mass bias effect was Isotopic Abundances of Iron (GBW04446, National Institute of Metrology, Beijing, China), a Fe isotopic certified reference material. All Fe isotopes were detected simultaneously in one sequence together with ^60^Ni as monitor for the correction of isobaric interferences of ^58^Ni on ^58^Fe. Mixture of Ar and H_2_ were used as collision gas to eliminate the interferences [37]. Under the optimized conditions, external precisions on the order of 0.01–0.03% (relative standard deviation, RSD), and 0.1–0.3% (RSD) were obtained for ^57^Fe/^56^Fe and ^58^Fe/^56^Fe, respectively. Enriched proportions of ^57^Fe/^56^Fe% and ^58^Fe/^56^Fe% were measured accordingly.

### Calculation of iron ^57^Fe absorption and infused ^58^Fe utilization

Non-heme iron absorption and utilization were assessed using stable isotope technique in which the incorporation of an oral ^57^Fe dose and intravenous ^58^Fe dose into erythrocytes of the subjects was measured, respectively. The following formulas were derived and used as described previously [38, 39]:

I.

Total oral absorption of ^57^Fe (mg) = [(Day 14 ^57^Fe:^56^Fe- baseline ^57^Fe:^56^Fe) / (Day 14 ^58^Fe:^56^Fe-

baseline ^58^Fe:^56^Fe)] × ^58^Fe administered (i.v. + orally) (mg) − ^57^Fe administered i.v. (mg)

^57^Fe absorption (%) = [total oral absorption of ^57^Fe (mg) / ^57^Fe administered (orally + i.v.) (mg)] x 100%

^57^Fe (mg) in ^58^Fe preparation = total dose of ^58^Fe preparation (mg) × concentration of ^58^ Fe

preparation (0.6933 mg/g) × ^57^Fe abundance in ^58^Fe (0.08826)

^58^Fe (mg) in ^57^Fe preparation = total dose of ^57^Fe preparation (mg) × concentration of ^57^ Fe

preparation (0.3297 mg/g) × ^58^Fe abundance in ^57^Fe (0.02106)

II.

Incorporation of ^58^Fe (%) = [(total circulation ^56^Fe mass × (Day 14 ^58^Fe:^56^Fe—baseline^58^Fe:^56^Fe) (mg)/ ^58^Fe administered i.v. (mg)] x 100%

Total circulating ^56^Fe mass (mg) = total iron mass × ^56^Fe abundance (0.9175) (mg)

Total iron mass (mg) = iron concentration (mg/g) × blood volume (BV) = Hb (g/L) ×amount of iron in 1 g Hb (3.47 mg/g) × BV (mL) / 1000 (mg). BV was calculated from body height (BH, cm) and body weight (BW, kg) using the formula published by Carlsen and Bruun [40], which was: BV (mL) = (45.2 + 25.3^(−0.0198 × DDW)^) × BW (kg), where DDW = 100 × (BW (kg)− 7.582^(0.01309 × BH (cm))^) / 7.582^(0.01309 × BH (cm))^

### Data analysis

Data were analyzed using SPSS software (SPSS Inc. Chicago, IL, USA). Among the data measured in this study, the values of serum ferritin and CRP and iron absorption might be considered to have abnormal distribution. To clarify this, a pilot Kolmogorov-Smirnov test was carried out. It was shown that the data of serum levels of CRP and SF, and ^57^Fe in both the rice and steamed buns groups had normal distribution (S1 and S2 Tables, all *P*>0.05). Other data also showed normal distribution (unpublished data). Therefore, these data were presented as mean ± standard deviation (SD), and were compared between the buns and rice groups using the Independent Sample *t*-test. Pearson correlation analysis was performed to analyze the relationships of iron absorption and utilization with iron nutritional status and inflammatory indicators. The level of significance was set at *P* < 0.05 (two-sides).

## Results

### Subject basic characteristics

The subjects’ age, height, weight, body mass index (BMI), and other iron indices are shown in Table 2. Overall, the subjects’ mean age and BMI were 21.6±1.5 years and 22.7±3.3 kg/m^2^, respectively. The subjects had good iron nutritional statuses (UIBC, SI, sTfR, SF, and Hb) at baseline; none had iron deficiency (SF level < 30 μg/L), early iron deficiency (sTfR >4.4 mg/L) or iron deficiency anemia (SF < 30 μg/L, and Hb < 130 g/L). None had inflammation according to the CRP level (mean CRP = 0.20±0.10 mg/L, which was far below the kit’s diagnostic standard of >5 mg/L). There were no significant differences in the basic characteristics (including age, weight, height, BMI) and serum level of CRP, UIBC, SI, sTfR, SF, and Hb at baseline between the rice and steamed buns groups (all *P* > 0.05) (Table 2).

**Table 2:** Subject basic characteristics.

			Rice group		Steamed buns group		
	Mean	SD	Mean	SD	Mean	SD	P value
**Age (years)**	21.6	1.5	22.1	1.2	21.0	1.7	0.16
**Weight (kg)**	67.8	12.7	65.2	11.9	70.4	13.0	0.23
**Height (cm)**	173.1	5.3	173.6	5.1	172.5	5.8	0.84
**BMI (kg/m**^**2**^**)**	22.7	3.3	21.7	3.3	23.7	3.3	0.14
**CRP (mg/L)**	0.20	0.10	0.20	0.08	0.10	0.08	0.19
**UIBC (μmol/L)**	22.8	11.3	21.7	10.2	24.2	11.2	0.42
**SI (μmol/L)**	16.9	7.8	19.0	7.6	14.8	8.9	0.25
**sTfR (mg/L)**	2.80	0.70	2.80	0.97	2.80	0.90	0.6
**SF (μg/L)**	63.8	25.6	83.6	24.5	52. 8	22.7	0.07
**Hb (g/L)**	140.6	8.6	139.9	8.4	141.4	7.4	0.73

BMI: body mass index, CRP: C-reactive protein, UIBC: unsaturated iron-binding capacity, SI: serum iron, sTfR: soluble transferrin receptor, SF: serum ferritin, Hb: hemoglobin

### Dietary sample test

The exact isotopic composition of the stable labels in the staple was shown in Table 3, including 96.727% ^57^Fe in ^57^Fe formulation and 90.966% ^58^Fe in ^58^Fe formulation, respectively. All major nutrients and enhancers and inhibitors of iron absorption in the meal samples were measured during the experimental period, and the subjects’ daily average nutrient intakes were calculated and shown in Table 4. The mean caloric supply of the total subjects was approximately 2400 Kcal, which is close to the 2013 recommended dietary energy intake for the young Chinese men with moderate physical activity levels (2600 Kcal) [15]. Particular, staple food provided about 1200 Kcal, accounting 53% of the total caloric supply. Among the total subjects, the percentages of energy from protein, fat, and carbohydrates were 18%, 28%, and 52%, respectively; all met the corresponding recommended values [15]. In addition, the molar ration of phytic acid to iron was 0.48:1 among all the subjects.

**Table 3:** Abundances of stable iron isotopes used in the experiment (%).

	^54^Fe	^56^Fe	^57^Fe	^58^Fe
^**57**^**Fe formulation**	0.010	1.157	**96.727**	2.106
^**58**^**Fe formulation**	0.014	0.194	8.826	**90.966**

**Table 4:** Daily average intakes of major nutrients, and enhancers and inhibitors of iron absorption.

			Rice group		Steamed buns group		
	Mean	SD	Mean	SD	Mean	SD	P value
**Calories (Kcal)**	2363.5	233.2	2348.8	266.7	2378.1	206.4	0.78
Iron (mg) *	12.8	2.1	11.3	1.3	14.3	1.5	<0.001*
**Zinc (mg)** *	9.2	1.7	10.3	1.4	8.1	0.9	<0.001*
**Calcium (mg)**	1179. 7	214.7	1161.9	275.3	1197.4	142.6	0.71
**Carbohydrate (g)**	306.5	42.6	309.5	49.9	303.4	37.3	0.74
**Fat (g)**	73.2	4.7	71.5	5.4	74.9	3.2	0.08
**Protein (g)**	111.3	10.6	108.8	10.8	113.8	10.2	0.27
**Dietary fiber (g)**	16.6	0.9	16.1	0.9	17.1	0.6	0.075
Ascorbic acid (mg)*	5.6	1.0	4.7	0.3	6.6	0.5	<0.001*
**Phytic acid (mg)**	72.6	36.6	71.0	30.9	74.2	43.1	0.84

* *P* < 0.001 represented significantly difference between rice group and steamed buns group.

There was no significant difference in the calories intake, and the content of carbohydrate, fat, protein, and dietary fiber between the rice and steamed buns groups (all *P*>0.05). The intakes of iron absorption inhibitors (including phytic acid and calcium) did not show significant difference between the groups either (P>0.05). However, the intake levels of ascorbic acid that promotes iron absorption were significantly higher in the steamed buns group than in the rice group (*P*<0.001) (Table 4).

Total iron intake was significantly lower in the rice group than in the steamed buns group (*P*<0.001). In contrast, total zinc intake was significantly higher in the rice group than in the steamed buns group (*P*<0.001).

### Non-heme iron absorption and biological utilization

During the 2-day experimental period, totally a mean of 6.49 mg ^57^Fe and 4.79 mg ^58^Fe was actually administered to each subject (Table 5). There was no significant difference in the actual doses of ^57^Fe and ^58^Fe in the meals, blood ^57^Fe (shown as ^57^Fe:^56^Fe) and ^58^Fe (shown as ^58^Fe:^56^Fe) levels at baseline, and ^57^Fe absorption and infused ^58^Fe utilization at 2 weeks after the experimental diet between the 2 groups (*P*>0.05).

**Table 5:** Erythrocyte iron isotopic enrichment and iron absorption (mean ± SD).

			Rice group		Steamed buns group		
	Mean	SD	Mean	SD	Mean	SD	P value
**Oral** ^**57**^**Fe dose (mg)**	6.49	0.18	6.47	0.23	6.51	0.11	0.632
**Oral** ^**58**^**Fe dose (mg)**^**1**^	0.141	0.003	0.141	0.005	0.142	0.002	0.632
**Dose** ^**58**^**Fe iv (mg)**	4.79	0.51	4.91	0.13	4.67	0.72	0.310
**Dose** ^**57**^**Fe iv (mg)**^**2**^	0.465	0.051	0.476	0.129	0.453	0.070	0.299
**Baseline** ^**57**^**Fe:**^**56**^**Fe**	0.02305	0.00000	0.02307	0.00002	0.02304	0.00001	0.745
^**57**^**Fe:**^**56**^**Fe at two weeks**	0.02359	0.00014	0.02360	0.00020	0.02350	0.00020	0.137
**Change in** ^**57**^**Fe:**^**56**^**Fe**	0.00052	0.00014	0.00050	0.00020	0.00040	0.00020	0.133
**Baseline** ^**58**^**Fe:**^**56**^Fe*	0.00305	0.00003	0.00304	0.00003	0.00306	0.00001	0.377
^**58**^**Fe:**^**56**^**Fe at two weeks**	0.0049	0.0003	0.0049	0.0002	0.0049	0.0004	0.894
**Change in** ^**58**^**Fe:**^**56**^**Fe**	0.0018	0.0003	0.0018	0.0001	0.0018	0.0003	0.953
^**57**^**Fe absorption (mg)**	0.1150	0.0731	0.1414	0.0847	0.0831	0.0482	0.061

^1^ Oral ^58^Fe dose means the dose of the mixed oral ^58^Fe from the oral ^57^Fe preparation.

^2^ Dose ^57^Fe iv means the dose of the mixed iv ^57^Fe from the iv ^58^Fe preparation.

The mean ^57^Fe absorption rate was 11% (range 4–18%). The rice group tended to have a higher oral ^57^Fe absorption rate (mean: 13%, range 6–20%) than the steamed buns group (mean: 8%, range 6–12%), although the difference was not significant (*P* = 0.061) (Table 6). The mean infused ^58^Fe utilization rate was 85% (range 77–93%); there was no significant difference between groups (rice group: mean 84%, range 78–90%; buns group: 85%, range 75–95%, *P* = 0.389) (Table 6).

**Table 6:** Subjects’ iron absorption and utilization rate.

			Rice group		Steamed buns group		
	Mean	SD	Mean	SD	Mean	SD	P value
^**57**^**Fe (oral) absorption rate**	11%	7%	13%	7%	8%	4%	0.061
^**58**^**Fe (iv) utilization rate**	85%	8%	84%	6%	85%	10%	0.389

### Correlations of iron absorption and utilization rate with iron nutritional status and inflammatory indicators

Pearson analysis showed that among the nutritional status and inflammatory indicators including UIBC, SF, sTfR, SI, Hb, and CRP, SF was significantly negatively correlated with ^57^FeSO_4_ absorption (*r* = −0.503, *P* = 0.017) and ^58^FeSO_4_ incorporation rate (*r* = −0.463, *P* = 0.02).

## Discussion

Studies have been reported the iron absorption from various diets such as American [23], Mexican [25], typical Latin American [19], a mixed American or Canadian [10], Danish [24] and British [41] diets. China has the largest (nearly 1.4 billion) population in the world, with the rice and wheat as the staple food in the Southern and Northern China, respectively. To determine the iron DRI value for Chinese population, it is rather important to observe the status of the dietary iron absorption from rice and wheat in Chinese population. With the help of a single-labeled stable isotope technique, Zhou et al. measured dietary iron absorption in 12 young Chinese Tibetan men (18–24 years old) and obtained an overall absorption rate of 13.4% [42], but that study just reflected the iron absorption in a very small portion of Chinese population because these subjects lived in the high altitude area and had great differences in diet patterns, iron nutritional status and genetic background from most of the other Chinese populations. Until now no available data can be used directly for the setting of Chinese iron DRIs, calling for the necessity of strictly estimation of the iron absorption from the typical whole Chinese diets in Chinese adult population. In this study, we investigated the non-heme iron absorption and biological utilization from typical whole diets in young Chinese urban men using a double-labeled stable isotope technique. In addition, given great difference in the diet patterns within China, we also observed if the iron absorption and utilization could be affected by the staple patterns of Southern and Northern China.

In this study, to correctly estimate the dietary iron absorption, typical whole Chinese diets instead of single meals were served for the subjects. It is very important to first formulate a representative experimental diet, which can collate data on inhibitors and enhancers of iron absorption in the diets. We designed typical whole Chinese diets on the basis of the results of the latest Chinese National Nutrition Survey [34] and cluster analysis. The total energy and percentages of protein, carbohydrate, and fat consumed by the subjects were all in accordance with the recommended dietary intakes for the Chinese population [15]. The molar ratio of phytic acid to iron can be used to estimate the negative effect of phytic acid on iron absorption [4, 43], and the ratio should be < 6:1 in composite meals with certain vegetables that contain ascorbic acid and animal meat as enhancers of iron absorption [4]. In our study, the molar ration of phytic acid to iron was 0.48:1, indicating that phytic acid does not influence the iron absorption from the whole diets in this study. In addition, iron status affects iron bioavailability, which needs to be assessed in combination, and inflammatory markers should be also measured simultaneously [8]. We thus strictly screened the subjects’ iron nutritional statuses in the current study. During the baseline screening of the subjects, we used the traditional criteria of Hb < 130 g/L and SF < 30 μg/L to rule out iron deficiency anemia. We also analyzed sTfR to exclude early iron deficiency (>4.4 mg/L) [2]. In addition, the impact of inflammation (defined as CRP > 5 mg/L) on relevant indicators was excluded. These results indicated that these subjects were healthy with good iron nutritional statuses.

Additional factors affecting the results of similar studies include the experimental isotope dose and administration method. In the present study, similar dose of ^57^FeSO_4_ was mixed with staple rice and steamed buns and ingested in 3 meals per day over 2 days in accordance with the energy distribution ratio. This strategy enabled the measurement of the representative dietary iron absorption under normal dietary conditions. We administered a mean oral ^57^Fe dose of 3.25 mg/d, which accounted for approximately 25% of the average daily dietary iron intake (12.8 mg). When coupled with the intravenous ^58^Fe dose of 2.4 mg/d, the total amount of labeled isotopic iron accounted for 44% ([3.25+2.4] mg/12.8 mg x 100%) of the total dietary iron intake. Stable isotope iron supplementation is reported to reach 30–60% of total dietary iron intake; at this level isotopes involved in will not interfere with the normal iron metabolism [44]. On the other hand, according to the fortification levels in China (14~26 mg iron/ kg rice or flour) [45], all the subjects in this study consumed average 450 g rice or flour, so the daily total labeled iron (3.25 mg ^57^Fe + 2.4 mg ^58^Fe = 5.65 mg Fe) in this study was less than the iron fortification level (6.3–11.7 mg [14–26 mg iron/ kg rice or flour x 0.45 kg]) in China, which would not influence the normal iron metabolism. In addition, we did not instruct the subjects to initially consume a low-iron diet and we distributed the stable isotope doses in food served at 6 meals over 2 days. This strategy is closer to actual dietary situations, thus avoiding overestimation of biological utilization. Collectively, this study was strictly designed.

The present result showed that the average iron intake in the basic staple diet was 12.8 mg, which is concordant with that reported by the 2000 Chinese Total Diet Study (national average iron intake among adult men: 13.03 mg, range 9.28–23.76 mg) (unpublished data). This value reflects the iron intake in the actual diet consumed by the Chinese population, and it is consistent with data from some countries with similar dietary habits to China (e.g. mean daily intake of 12.7–15.0 mg iron for Japanese men [46] but unsurprisingly inconsistent with other countries with different dietary habits (e.g. mean daily total dietary iron intake of 13.5 mg for British men) [47]. We designed two sub-groups with steamed buns or rice their staple food respectively according to the different dietary habits of Northern and Southern China. The iron intake of the steamed buns group was significantly higher than that of the rice group, which might be because the steam buns group subjects consumed more iron-rich animal tissues (including stir-fried chicken breast, braised beef and stir-fried squid). However, since the isotopic ^57^Fe labels in the meals were similar among all these subjects, the final ^57^Fe absorption from the whole diet did not show significant difference between these two sub-groups.

In this study, in addition to the staple food, some non-staple foods were similarly provided for the subjects. However, probably due to the difference of the subjects’ dietary habits, the non-staple foods were not similarly taken by the subjects. Some kinds of foods relatively rich in ascorbic acid were taken less by rice group subjects than by streamed buns group subjects. As a result, the intake levels of ascorbic acid were significantly lower in the rice group than in the steamed buns group. Although ascorbic acid is an enhancer of iron absorption, generally, there is no significant difference in the non-heme iron absorption and utilization between the two groups, implying that difference of the levels of single enhancer or inhibitor might not be able to substantially influence the non-heme iron absorption and utilization.

Iron absorption represents the percentage of the absorbed iron (from the dietary iron) that enters the gastrointestinal mucosa cells through gastrointestinal tract, which is calculated as the incorporation of oral ^57^Fe into erythrocytes. It was reported that the mean non-heme iron absorption from whole diets in other countries (such as USA, Europe, and Mexico) greatly varied from 0.7 to 22.9% [21–25]. The present study demonstrated that the mean dietary non-heme iron absorption in young Chinese men with typical whole diets was 11±7%. Kalasuramath et al. showed 8.3% of the mean iron absorption from rice and 11.2% from wheat-based meals in healthy young Indian women with iron deficiency [48]. Moretti et al. reported the mean iron absorption was 11.6% from rice meals fortified with ferrous sulfate in young women and 3.2% from wheat-based meal [49]. Our study showed the dietary non-heme iron absorption in rice group was higher than in wheat-based streamed bund group (13±7% versus 8±4%, *P* = 0.061), which is consistent with Moretti et al.’ study [49] while is interestingly different from Kalasuramath et al.’s study [48].

Iron utilization shows the ratio of the iron that is utilized by body to synthesize the iron–containing biomolecules (mainly hemoglobin) among the absorbed iron from dietary iron during a period. It is usually expressed as the incorporation ratio of the absorbed iron into erythrocytes or hemoglobin of the subjects, which is calculated as the incorporation of iv ^58^Fe into erythrocytes in this study. In this study, the overall infused iron utilization rate at 14 days after intake was 85±8%, which is similar to the results of foreign studies (80–93%) [39, 50]. We administered two different stable isotope tracers orally and intravenously to directly measure the incorporation of the infused iron into erythrocytes, which helped distinguish dietary iron absorbed by the intestinal tract and utilized systemically [31–33]. In contrast, single-labeled isotope method cannot directly obtain this data [42]. Thus, the results will be beneficial to clarify dietary iron absorption and utilization in the human body.

Our results demonstrated that iron store (SF level) was negatively correlated with iron absorption, which is consistent with Dainty et al.’s result [47, 51]. The iron bioavailability factor for DRIs thus needs to be practically relevant and for a well-defined iron status. There lacks agreement with regard to the iron status on which the estimation of iron absorption values should be based. However, it is commonly considered that the iron absorption would be attained from subjects who have low iron stores but are not anemic [47]. Under this condition, iron absorption can be maximally up-regulated without any impairment of normal physiological processes. It was suggested that the absorption efficiency should be based on subjects with the SF concentration of 15 μg/L [10]. This will lead to a higher bioavailability and lower dietary iron intake recommendation, but still ensure that subjects with low iron stores will absorb enough iron to meet their demands. Yet whether this SF level is sufficient to meet the needs of individuals with adequate iron stores is unclear [4]. Researchers from different countries have proposed specific SF cut-offs for domestic populations for the diagnosis of iron deficiency, on which should be based to estimate the iron absorption efficiency. For example, Nelson et al. [52] and Sharma et al. [53] supposed a cut-off level of 30 μg/L for SF. A meta-analysis revealed that the determination of iron deficiency using a cut-off level of 25 or 30 μg/L achieved the greatest accuracy and overall effectiveness [54]. In the present study, the non-heme absorption and utilization in young Chinese adult urban men were estimated on the condition that SF level was measured as 63.8±25.6 μg/L using the detection Kit with the reference range of 30–400 μg/L for adult men with normal SF levels. Since the SF concentration is strongly dependent on the techniques and also on the used assay/Kit, we should adjust the SF level when use this data for iron DRIs setting.

Generally, the basic characteristics (age, weight, height, BMI), inflammatory parameters, iron status, most of the daily average intakes of major nutrients and enhancers and inhibitors of iron absorption (including calories, calcium, carbohydrate, fat, protein, dietary fiber, phytic acid), oral ^57^Fe dose and iv administrated ^58^Fe dose of the subjects in the rice and streamed buns groups were almost consistent without significant difference (all *P*>0.05). There was no significant difference in the iron absorption from dietary iron and absorbed iron utilization between the two groups (both *P*>0.05). These results indicate the iron absorption and utilization from typical whole Chinese staple food are not influenced by the representative patterns of the staple food in China.

We recruited young men, the standard population for the setting of DRIs, to observe the non-heme iron absorption and utilization from typical whole Chinese dietary (including rice as the representative staple food in Southern China and wheat-based streamed buns as the representative staple food in Northern China). For women, Pizarro et al. showed mean iron absorption of 10.5% (4.1–27.0%) from the wheat flour bread fortified with ferrous sulfate [55]. Moretti et al. showed from the rice meals fortified with ferrous sulfate, the iron absorption in young women was 11.6% [49]. It was reported that there was no significant difference in the iron bioavailability from meals between male and female [56]. The iron utilization between male and female is unknown. A parallel study to observe the non-heme iron absorption and utilization from typical Chinese staple food in women is being performed by our group, whereby the non-heme iron absorption and utilization from typical Chinese staple food in male versus female will be directly compared.

Iron absorption varies, which depends on the food sources, subject’s nutritious status, etc. It was shown that the mean iron absorption from the dal was low as 2.20 ± 3.40% in nonpregnant women [57]. Non-heme iron absorption from the beef meal in children was significantly greater than from the soy meal [58]. A report showed mean iron absorption was low (4.6%) from the millet, but was increased from rice (8.3%) and wheat (11.2%)-based meals in the iron deficiency healthy young Indian women [48]. Iron absorption from a rice-based meal was low in iron replete (2.7%) but was higher in iron deficiency anemia Indian adult women (2.7% versus 8.3%) [59]. Further studies need to be carried out to observe the iron absorption and utilization from other staple foods (e.g. maize, potato) and in subjects with different health status (e.g. iron-replete, iron-deficient) in China.

There are some limitations of this study. First, this study has a relatively short feeding period with 6 meals. Observations during a longer experimental period are needed to verify the present results. Second, since there were neither data with regard to the dietary iron absorption from rice and wheat in Chinese population nor pilot data of the difference in the dietary iron absorption between rice and wheat in young Chinese men, it was not easy to estimate the minimum sample size for this randomized controlled study. In this study, the determination of the sample size was based on some previous studies involving double-labeled iron stable isotopes [22, 23, 55, 60]. The sample size of 22 in this study appears to be small. However, the double-labeled stable isotopic technique used in this study involves labeling with two kinds of stable isotopes, which is expensive for the analysis. As a result, such studies usually involve rather limited number (10 or a bit more than 10 subjects) [22, 23, 55–57, 60]. The subject number in our study is comparable to and even a bit more than in the above studies. Nevertheless, in the next randomized controlled study, more subjects will be included and the minimum sample size will be estimated on the basis of the present study to validate these results. Third, it is known that mutations in several genes such as *HFE*, *Hjv*, *HAMP* and *FPN* result in hereditary hemochromatosis (HH), a disorder that causes excess high iron absorption [26, 27]. We did not clarify whether or not these gene mutations occurred in the subjects in this study, however, it is a fact that HH is rare in Chinese Han population, which is related to the low incidence of the HFE gene mutations [27–30]. Moreover, these subjects in this study were healthy with good iron nutritional statuses. Therefore, the possibility of the existence of HFE gene mutations could be excluded. Nevertheless, next we will detect the gene mutations associated with HH to fully exclude the influence of the mutations of these HH genes on the dietary iron absorption. Next studies will also be focused on investigating the iron absorption and utilization among different populations, especially those from high incidence areas of anemia and those (e.g. pregnant women, children) prone to have anemia.

## Conclusion

We strictly assessed the non-heme iron absorption and utilization rates from typical whole Chinese diets in young Chinese healthy adult urban men, which was not affected by the representative staple food patterns of Southern and Northern China. This study will provide the basis for the setting of iron DRIs for Chinese inhabitants. Further studies with longer observation period and more number of subjects, and studies investigating iron absorption and utilization from other staple foods, in subjects with different health status, and in different populations in China will be performed to validate these results.

## Supporting Information

S1 Table Kolmogorov-Smirnov test for Rice group. (DOC)

S2 Table Kolmogorov-Smirnov test for Steamed buns group. (DOC)
