# Supplementary material for: Non-Heme Iron Absorption and Utilization from Typical Whole Chinese Diets in Young Chinese Urban Men Measured by a Double-Labeled Stable Isotope Technique
Source: PLoS One. 2016 Apr 21;11(4):e0153885. doi: 10.1371/journal.pone.0153885 (PMC4839665; doi:10.1371/journal.pone.0153885)
Supplement: S1 Table — (DOC) [file pone.0153885.s001.doc]

| **S1 Table. Kolmogorov-Smirnov test for Rice group** | | | | |
| --- | --- | --- | --- | --- |
|  | | CRP mg/L | SF ug/L | ^57^Fe absorption |
| N | | 11 | 11 | 11 |
| Normal parameter ^a, b^ | Mean | 0.1191 | 83.5818 | 0.13161613533805 |
|  | SD | 0.08312 | 24.57632 | 0.078841166155735 |
| Most extreme difference | Absolute value | 0.227 | 0.174 | 0.137 |
|  | Positive | 0.180 | 0.174 | 0.137 |
|  | Negative | -0.227 | -.0161 | -0.091 |
| Kolmogorov-Smirnov Z | | 0.752 | 0.578 | 0.453 |
| Asymptotic significance (two-side) | | **0.625** | **0.892** | **0.986** |
| a. Normal distribution | | | | |
| b. Calculated from data | | | | |
