# Supplementary material for: Non-Heme Iron Absorption and Utilization from Typical Whole Chinese Diets in Young Chinese Urban Men Measured by a Double-Labeled Stable Isotope Technique
Source: PLoS One. 2016 Apr 21;11(4):e0153885. doi: 10.1371/journal.pone.0153885 (PMC4839665; doi:10.1371/journal.pone.0153885)
Supplement: S2 Table — (DOC) [file pone.0153885.s002.doc]

| **S2 Table. Kolmogorov-Smirnov test for Steamed buns group** | | | | |
| --- | --- | --- | --- | --- |
|  | | CRP mg/L | SF ug/L | ^57^Fe absorption |
| N | | 11 | 11 | 11 |
| Normal parameter ^a,b^ | Mean | 0.1045 | 52.8200 | 0.07748379094079 |
|  | SD | 0.08202 | 22.65260 | 0.044664488033006 |
| Most extreme difference | Absolute value | 0.205 | 0.192 | 0.187 |
|  | Positive | 0.172 | 0.192 | 0.187 |
|  | Negative | -0.205 | -0.139 | -0.146 |
| Kolmogorov-Smirnov Z | | 0.680 | 0.638 | 0.619 |
| Asymptotic significance (two-side) | | **0.743** | **0.810** | **0.838** |
| a. Normal distribution | | | | |
| b. Calculated from data | | | | |
